# A Flavonoid-Rich Extract of Mandarin Juice Counteracts 6-OHDA-Induced Oxidative Stress in SH-SY5Y Cells and Modulates Parkinson-Related Genes

**DOI:** 10.3390/antiox10040539

**Published:** 2021-03-30

**Authors:** Santa Cirmi, Alessandro Maugeri, Giovanni Enrico Lombardo, Caterina Russo, Laura Musumeci, Sebastiano Gangemi, Gioacchino Calapai, Davide Barreca, Michele Navarra

**Affiliations:** 1 Department of Pharmacy-Drug Sciences, University of Bari “Aldo Moro”, 70125 Bari, Italy; santa.cirmi@uniba.it; 2 Department of Chemical, Biological, Pharmaceutical and Environmental Sciences, University of Messina, 98168 Messina, Italy; amaugeri@unime.it (A.M.); carusso@unime.it (C.R.); laura.musumeci@unime.it (L.M.); davide.barreca@unime.it (D.B.); 3 Fondazione “Prof. Antonio Imbesi”, 98123 Messina, Italy; 4 Department of Clinical and Experimental Medicine, University of Messina, 98125 Messina, Italy; sebastiano.gangemi@unime.it; 5 Department of Biomedical and Dental Sciences and Morphofunctional Imaging, University of Messina, 98125 Messina, Italy; gioacchino.calapai@unime.it

**Keywords:** neurodegenerative diseases, Parkinson’s disease, mandarin juice, *Citrus reticulata*, 6-OHDA, neuroprotection, SH-SY5Y, oxidative stress, natural products

## Abstract

Parkinson’s disease (PD) is a degenerative disorder of the nervous system due to unceasing impairment of dopaminergic neurons situated in the substantia nigra. At present, anti-PD drugs acting on dopamine receptors are mainly symptomatic and have only very limited neuroprotective effects, whereas drugs slowing down neurodegeneration of dopaminergic neurons and deterioration of clinical symptoms are not yet available. Given that, the development of more valuable pharmacological strategies is highly demanded. Comprehensive research on innovative neuroprotective drugs has proven that anti-inflammatory and antioxidant molecules from food sources may prevent and/or counteract neurodegenerative diseases, such as PD. The present study was aimed at the evaluation the protective effect of mandarin juice extract (MJe) against 6-hydroxydopamine (6-OHDA)-induced SH-SY5Y human neuroblastoma cell death. Treatment of differentiated SH-SY5Y cells with 6-OHDA brought cell death, and specifically, apoptosis, which was significantly inhibited by the preincubation with MJe through caspase 3 blockage and the modulation of p53, Bax, and Bcl-2 genes. In addition, it showed antioxidant properties in abiotic models as well as in vitro, where it reduced both reactive oxygen and nitrogen species induced by 6-OHDA, along with restored mitochondrial membrane potential, and prevented the oxidative DNA damage evoked by 6-OHDA. Furthermore, MJe restored the impaired balance of SNCA, LRRK2, PINK1, parkin, and DJ-1 gene levels, PD-related factors, caused by 6-OHDA oxidative stress. Overall, these results indicate that MJe exerts neuroprotective effects against 6-OHDA-induced cell death in SH-SY5Y cells by mechanisms involving both the specific interaction with intracellular pathways and its antioxidant capability. Our study suggests a novel possible strategy to prevent and/or ameliorate neurodegenerative diseases, such as PD.

## 1. Introduction

Parkinson’s disease (PD) is an aging-related neurodegenerative disease (ND) whose characteristics are progressive aggregation of α-synuclein in surviving neurons and selective death of dopaminergic neurons in the substantia nigra, particularly its pars compacta [1]. PD shows tremor at rest, rigidity, bradykinesia, slowed movements, and postural instability. The Global Burden of Diseases 2016 has gathered the evidence on neurological disorders, showing that PD had an incidence of 1 million cases worldwide, of which more than 150,000 in the EU, and PD-related deaths were more than 340,000 globally, with 60,000 just in the EU [2]. Although the actual triggers of PD remain undefined, the link between oxidative stress, mitochondrial impairment, protein misprocessing, and genetic variation is pivotal in the pathogenesis of the disease [3].

In the unceasing search of innovative neuroprotective drugs, anti-inflammatory and antioxidant molecules from dietary sources have been suggested to prevent and/or counteract NDs, such as PD [4,5,6]. In this context, nutraceuticals along with food supplements have been proven to provide neuroprotective effects in several experimental models, and their use as substitute to synthetic drugs or in combination with them is supported by their ability to hinder oxidative stress as well as to interact with intracellular pathways involved in NDs [4,7,8]. These properties might be ascribed to the presence of polyphenolic compounds, abundantly present in fruits, vegetables, cereals, and beverages. The main class of polyphenols is flavonoids, whose major dietary sources are fruits, especially citrus, vegetables, tea, coffee, and red wine. They possess a remarkable spectrum of biological activities, such as antioxidant, free radical scavenging, metal ion chelating, vasoprotective, hepatoprotective, anti-cancer, anti-infective and anti-inflammatory [9,10,11,12,13].

Based on this, we investigated whether a flavonoid-rich extract from *Citrus reticulata* Blanco (mandarin; MJe) protects differentiated SH-SY5Y cells from 6-hydroxydopamine (6-OHDA)-induced neurotoxicity and explored its mechanism of action.

## 2. Materials and Methods

### 2.1. Drug

*Citrus reticulata* Blanco fruits were harvested from crops located in Sicily (Italy). The flavonoid fraction of mandarin juice (MJe) was provided by the company “Agrumaria Corleone” (Palermo, Italy). The extract was obtained by passing the mandarin juice through columns equipped with flavonoid-adsorbent resins, which were then eluted. The extract was centrifuged for 15 min at 6000 rpm and then spray-dried. One hundred milliliters of MJe yielded 109 mg of dry extract. Aliquots of MJe were stored at −20 °C and defrosted just before use.

### 2.2. Chemical Characterization of MJe

#### 2.2.1. Reagents and Standard Solutions

HPLC-grade acetonitrile and DMSO were obtained by Sigma-Aldrich (St. Louis, MO, USA). Vicenin-2, hesperidin, eriocitrin, narirutin, sinensetin, nobiletin, and tangeretin were supplied from Extrasynthèse (Genay, France). Lucenin-2 4′-methylether and orientin 4′-methyl ether were separated from *Citrus limetta* and *Citrus bergamia* [14,15] and used as standards. The Iso-Disc P-34, 3 mm diameter PTFE membrane (0.45 μm pore size) was from Supelco (Bellefonte, PA, USA). All the other reagents and chemicals employed in this study were of analytical grade and were purchased from Sigma (St. Louis, MO, USA).

#### 2.2.2. Sample Preparation

A solution of DMSO/H_2_O (1:1) was added to the lyophilized powder to obtain a final concentration of (10.0 mg/mL), and the mixture was centrifuged for 5 min at 3200 rpm. The supernatant liquid was filtered through an Iso-Disc P-34, 3 mm diameter PTFE membrane with a 0.45 μm pore size (Supelco, Bellefonte, PA, USA) and utilized for HPLC-DAD separation.

#### 2.2.3. RP-DAD-HPLC Separation and Identification

Reverse phase-diode array detector-high performance liquid chromatography (RP-DAD-HPLC) was performed using a Shimadzu system (Shimadzu Ltd., Canby, OR, USA), consisting of a LC-10AD pump system, a vacuum degasser, a quaternary solvent mixing, an SPD-M10A diode array detector, and a Rheodyne 7725i injector. The separation of each compound was performed on a 250 × 4.6 mm i.d., 5 mm Discovery C18 column, supplied by Supelco (Bellefonte, PA, USA), equipped with a 20 × 4.0 mm guard column. The column was placed in a column oven set at 30 °C. The injection loop was 20 µL, and the flow rate was 1.0 mL/min. The mobile phase consisted of a linear gradient of acetonitrile in H_2_O as follows: 5–20% (0–15 min), 20–30% (15–20 min), 30–50% (20–30 min), 50–100% (30–35 min), 100% (35–40 min), 100–5% (40–50 min), and 5% (50–60 min). UV spectra were recorded between 200 and 600 nm, and simultaneous detection by diode array was performed at 278, 310, and 325 nm. Each sample was tested three times and gave superimposable chromatograms. Peak identification was performed by matching retention time and UV spectra against reference compounds and spiking the samples with pure reference compounds. The calibration lines were obtained using known concentrations of pure compounds and selected to match the concentration present in the tested sample. Quantitative analysis was carried out by integration of the areas of the peaks from the chromatogram at 280 and 325 nm for flavanone and flavone derivatives, respectively. The calibration curves were constructed, and linear regression equations were obtained by plotting the ratios of compounds’ peak areas to the peak areas of the external standard, against the known concentrations of pure compounds.

#### 2.2.4. Acid Hydrolysis

Hydrolysis was performed on MJe according to already published procedure [16]. Briefly, 10 mL of HCl (6 M) in a methanol (25 mL)/water (10 mL) solution was added to 5 mL of MJe to obtain a solution of 1.2 M HCl in 50% aqueous methanol. As an antioxidant, we added ascorbic acid (50 mg). After refluxing at 90 °C for 20 h under constant stirring, the solution was cooled at room temperature, the solvents evaporated under reduced pressure, and the residue suspended in 10 mL water/DMF (1:1). The mixture was filtered through an Iso-Disc P-34 membrane and analyzed by HPLC.

### 2.3. Cell Culture and Treatment

Experiments were carried out using the SH-SY5Y human neuroblastoma cell line originally from ATCC (Rockville, MD, USA). Their differentiation was performed with 10 µM retinoic acid (RA; Sigma–Aldrich, Milan, Italy) in MEM/Ham’s F12 medium supplemented for 5 days [17]. All reagents were from Gibco (Life Technologies, Monza, Italy). The stock solution of MJe 400 mg/mL was prepared in DMSO that was employed, upon further dilution in culture medium, to obtain the working concentrations. The same percentages of DMSO present in these dilutions served as vehicle controls that were tested in each of the following experiments to confirm that no effect was induced by the solvent (data not shown).

### 2.4. Cytotoxicity Assays

Cell viability was evaluated by the 1-(4,5-dimethylthiazol-2-yl)-3,5-diphenylformazan (MTT) test as reported by Morisi and collaborators [18]. Cells were seeded into 96-well plates at density of 5 × 10^4^ cells/well and left to attach overnight. Then, cells were treated with MJe at various concentrations (from 0.001 to 1 mg/mL) for 1 h. As stressor, 6-OHDA 50 µM (Sigma–Aldrich, Milan, Italy) was added for additional 24 h [17]. Afterwards, the plates were centrifuged, supernatants were removed, and fresh media without phenol red containing 0.5 mg/mL of MTT (Sigma-Aldrich) was added to each well. Plates were put in the incubator for an additional 4 h. Then, formazan crystals were solubilized in 100 μL HCl/isopropanol 0.1 N. The absorbance was spectrophotometrically quantified through iMark™ microplate reader (Bio-Rad Laboratories, Milan, Italy) at a wavelength of 570 nm. The viability was determined as percentage of viable cells in treated cultures compared to those in untreated ones.

Cell death was assessed by the trypan blue (0.4% *w/v*; TB) exclusion test. Cells were seeded onto 6-well plates at a density of 1 × 10^4^ cells/well for 24 h and then treated with MJe (0.001–1 mg/mL) for 1 h before exposure to 6-OHDA (50 μM) for another 24 h. Then, cells were detached and stained with trypan blue dye before proceeding with cell count [19,20]. Dead cells were reported as percentage of stained (nonviable) vs. total cells counted.

### 2.5. Detection of Apoptotic Cell Death and Caspase-3 Enzymatic Activity

The protective effect of MJe against 6-OHDA-induced cell death was analyzed by fluorescence-activated cell sorting (FACS), using the Annexin V-fluorescein isothiocyanate (FITC)/propidium iodide (PI) staining [21], a method employed to discriminate the living status of cells.

SH-SY5Y were seeded similarly to TB assay, but pretreated for 1 h with MJe (0.1 and 0.5 mg/mL) and then exposed to 50 μM 6-OHDA for another 24 h. Next, cells were collected and processed as already reported [21]. Finally, samples were run on a Novocyte 2000 cytofluorimeter (ACEA Biosciences Inc., San Diego, CA, USA).

Caspase 3 enzymatic activity was measured using a commercial kit (AbCam, Cambridge, UK). SH-SY5Y cells were differentiated in 100 mm petri dishes (1.5 × 10^6^ cells) and then treated for 6 h, as explained above. Then, according to the manufacturer’s instructions, the analysis was carried out on cell lysates [17]. Absorbance was measured at 405 nm by a microplate spectrophotometer.

### 2.6. Real-Time PCR Analysis

For the evaluation of MJe treatment on the expression of genes encoding for regulatory proteins of apoptosis, SH-SY5Y cells were seeded and treated as for the apoptosis evaluation. Afterwards, total RNA of treated or untreated SH-SY5Y cells was extracted and reverse transcribed following procedures of Currò and collaborators [22]. Messenger RNA levels were analyzed by quantitative real-time PCR, using SYBR green as a fluorescent probe. The analysis was carried out on a 7300 Real-Time PCR System (Applied Biosystems). β-Actin was used as housekeeping control, and a standard dissociation stage was included to assess primer specificity. Data were collected and analysed using the 2^−∆∆CT^ relative quantification method [22]. Values are presented as fold change relative to untreated cells. The primer sequences used for real-time PCR are listed in Table 1.

### 2.7. Evaluation of MJe Anti-Oxidant Activity through Abiotic Assays

The total phenolic content of MJe was evaluated through the Folin–Ciocalteu assay and its total antioxidant activity through the oxygen radical absorbance capacity (ORAC) assay, following Ferlazzo and co-workers [23]. The 2,2-Diphenyl-1-picrylhydrazyl (DPPH•) was employed to test the radical scavenging activity of our extract, in accordance to Lombardo and co-workers [24]. The reducing power of MJe was determined according to Ferlazzo and collaborators through the potassium ferricyanide reducing power assay [25]. All tests were repeated three times, and the results are expressed as means ± SEM. As standards, we employed gallic acid, Trolox, and ascorbic acid, in relation to the assay performed.

### 2.8. Measurement of Glutathione, Catalase and Superoxide Dismutase Activity

Catalase (CAT) and superoxide dismutase (SOD) activities and glutathione (GSH) content were measured using commercial assay kits (AbCam). SH-SY5Y cells were plated at density of 5 × 10^5^ cells/well in 6-well plates and were treated with MJe 24 h later at a concentration 0.1 and 0.5 mg/mL for 1 h. Then, 50 µM of 6-OHDA was added for additional 24 h. Then, the assays were conducted according to the manufacturer’s protocols on cell lysates. The absorbance was recorded by an iMark™ microplate reader (Bio-Rad Laboratories) at 450 nm for SOD, 570 nm for CAT, and 405 nm for GSH [25,26].

### 2.9. Determination of Reactive Oxygen Species (ROS) and Mitochondrial Membrane Potential (ΔΨm)

Reactive oxygen species (ROS) and mitochondrial membrane potential (ΔΨm) were measured fluorometrically, as oxidative stress-related biomarkers. In both assays, cells were seeded on 96-well plates (5 × 10^4^ cells/well) and treated as explained for caspase 3 activity (see Section 2.5).

ROS were quantified using probe 2′,7′-dichlorodihydrofluorescein diacetate (DCFH-DA) 25 μM (Sigma-Aldrich) [23] while variation of ΔΨm were estimated by measuring Rhodamine 123 incorporation (R123; Sigma-Aldrich), as reported by [25]. The fluorescence was recorded by a microplate reader (POLARstar Omega, BMG Labtech, Ortenberg, Germany) at 485 nm excitation and 535 nm emission for DCFH-DA and 488 nm excitation and 525 nm emission for R123.

### 2.10. Cytofluorimetric Evaluation of 8-oxo-dG

Oxidative DNA damage was assessed as levels of 8-Oxo-2’-deoxyguanosine (8-oxo-dG) employing the FITC-labelled avidin probe, which is highly affine to 8-hydroxyguanine (8-OH-Gua), being structurally similar to biotin. Briefly, cells were permeabilized with methanol at −20 °C for 15 min and incubated with avidin-FITC conjugate (0.2 μM) at 37 °C for 1 h. The fluorescence was recorded cytofluorimetrically with Novocyte 2000 cytofluorimeter at 495 nm excitation and 520 nm emission (ACEA Biosciences Inc.) [23].

### 2.11. Determination of NO Accumulation in SH-SY5Y Culture Supernatant

The production of nitric oxide (NO) was assayed by a colorimetric commercial kit (Sigma-Aldrich). In a 6-well plate, 5 × 10^5^ cells/well were preincubated with MJe for 1 h, and then treated with 50 µM 6-OHDA for 24 h. Supernatants were collected and processed as described by Ferlazzo and colleagues [17]. Absorbance was recorded at a wavelength of 540 nm by an iMark™ microplate reader (Bio-Rad Laboratories).

### 2.12. Statistical Analyses

One-way analysis of variance (ANOVA) was used to interpret the data. Multiple comparisons of the means of the groups were performed using the Tukey–Kramer test (SigmaPlot Version 12.0, Systat Software, San Jose, CA, USA).

## 3. Results

### 3.1. Chromatographic Analysis of MJe

**Figure 1:**
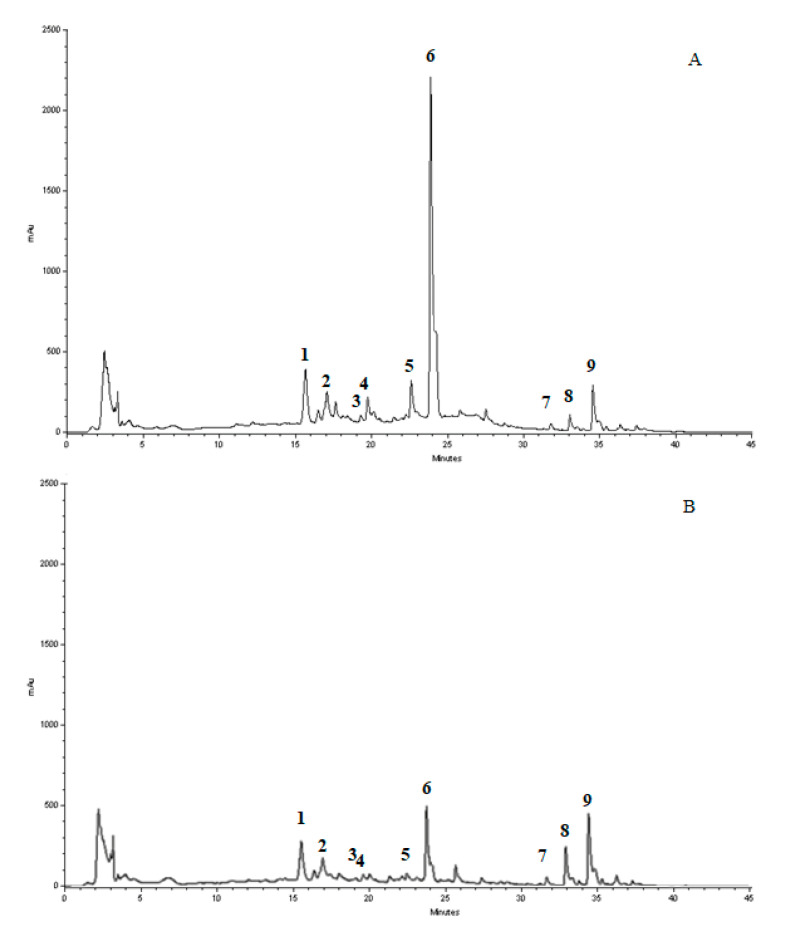
Reverse phase-diode array detector-high performance liquid chromatography (RP-HPLC-DAD) separation of the compounds present in the lyophilized powder registered at 280 (**A**) and 325 nm (**B**). Vicenin-2 (1), lucenin-2 4′-methyl ether (2), orientin4′methylether (3), eriocitrin (4), narirutin (5), hesperidin (6), sinensetin (7), tangeretin (8), nobiletin (9). Peak identification was performed by matching retention time and UV spectra against commercially available reference compounds and spiking the samples with pure reference compounds.

The identification of the compounds present in the lyophilized powder was performed by RP-DAD-HPLC. The preliminary inspection of chromatographic separation at 280 and 325 nm let us easily identify and discriminate the presence of flavanone and flavone basic skeletons in the lyophilized powder. Both classes of flavonoids showed maxima of absorption (called band II) in the 240–280 nm range, but only flavones had a further well-defined absorption maximum in the 300–380 nm range (called band I). The comparison, together with inspection of UV spectra recorded in correspondence to each chromatographic peak, showed that compounds 1–3 and 7–9 possessed a flavone skeleton, whereas 4–6 belonged to the flavanone class (Figure 1). Moreover, the inspection of chromatogram recorded after treatment with aqueous HCl (data not shown) indicated that compounds 1–3 were resistant to acidic hydrolysis, whereas the remaining compounds were not resistant, suggesting the presence of C-linked saccharide moieties in the former, while the latter were O-glycoside compounds. Taking into consideration the retention time, UV spectra and spiking the samples with pure reference compounds, the main peaks of the chromatogram have been identified as vicenin-2 (1), lucenin-2 4′-methyl ether (2), orientin4′methylether (3), eriocitrin (4), narirutin (5), hesperidin (6), sinensetin (7), tangeretin (8), and nobiletin (9). The flavonoid profile identified after the separation of compounds present in the lyophilized powder was dominated by the presence of the flavanone hesperidin, which was by far the most abundant component (about 353.6 mg/g), followed by a significant amount of di-C-glucosyl flavone (6,8-di-C-glucosyl-apigenin, about 55.2 mg/g) and another rutinoside (narirutin), although in smaller amount (about 47.8 mg/g). The quantitative analysis of all the identified flavonoids is depicted in Table 2.

### 3.2. MJe Prevents 6-OHDA Induced SH-SY5Y Cell Death

**Figure 2:**
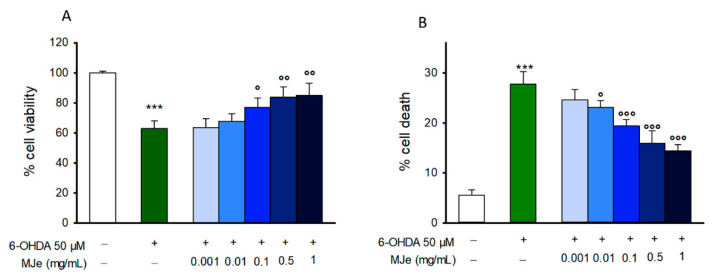
Protective effect of mandarin juice extract (MJe) against 6-hydroxydopamine (6-OHDA)-induced cytotoxicity. Cell viability was detected with the 1-(4,5-dimethylthiazol-2-yl)-3,5-diphenylformazan (MTT) assay (**A**). The viability variation was calculated as percentage of viable cells in treated cultures relative to those in untreated ones. Cell death was estimated by the trypan blue (TB) assay and expressed as percentage of nonviable (blue stained) cells relative to total counted cells (**B**). Results are displayed as mean ± standard error of the means (SEM) from three independent experiments in eight replicates (MTT; N = 24) or in triplicate (TB; N = 9). *** *p* < 0.001 vs. ctrl; ° *p* < 0.05, °° *p* < 0.01 and °°° *p* < 0.001 vs. 6-OHDA 50 µM.

To evaluate the potential neuroprotective effect of MJe, SH-SY5Y cells were preincubated with MJe and then exposed to 6-OHDA before the assessment of cell viability. The incubation of SH-SY5Y cells with 6-OHDA reduced cell viability by 37 ± 5.1% compared to that of control cells (*p* < 0.001; Figure 2A), whereas pretreatment with MJe at concentration of 0.1, 0.5 and 1 mg/mL restored cell viability up to 77 ± 6.1, 83 ± 6.9, and 85 ± 7.9%, respectively (*p* < 0.05 and *p* < 0.01 vs. 6-OHDA-injured cells; Figure 2A). Data of TB assay followed the same pattern of those of MTT test (Figure 2B). In particular, 6-OHDA increased cell death by 28 ± 2.5%, whereas 0.01, 0.1, 0.5, and 1 mg/mL MJe maintained it to 23.1 ± 1.3, 19.4 ± 1.2, 15.9 ± 2.6, and 14.4 ± 2.0%, respectively (*p* < 0.05 and *p* < 0.01 vs. 6-OHDA-exposed cells; Figure 2B).

### 3.3. MJe Reduces the Apoptotic Cell Death Induced by 6-OHDA

**Figure 3:**
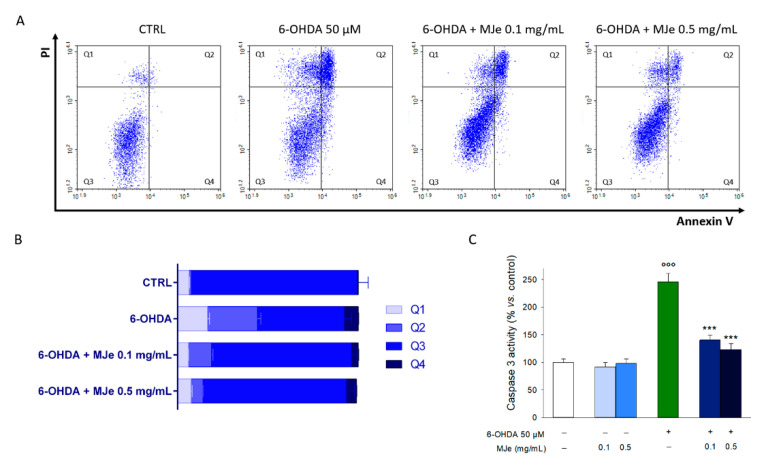
MJe mitigated the apoptosis induced by 6-OHDA in SH-SY5Y cells. (**A**) Evaluation of apoptosis was performed cytofluorimetrically by the Annexin V/PI test. Representative Annexin V vs. PI dot plots are shown, where necrotic, late apoptosis, viable cells, and early apoptosis cells are in Q1, Q2, Q3 and Q4, respectively. (**B**) The histogram shows the percentage of cells in each quadrant, representing the mean ± SEM of three different experiments in triplicate (N = 9). (**C**) Data of caspase 3 activity are presented as the mean of three experiments ± SEM in triplicate (N = 9). °°° *p* < 0.001 vs. control; *** *p* < 0.001 vs. 6-OHDA 50 µM.

The cytoprotecting activity elicited by MJe was also evaluated by flow cytometry through an Annexin V-FITC/PI assay. As shown in Figure 3A,B, the incubation of SH-SY5Y cells with 6-OHDA for 24 h augmented the percentage of cells in early (8 ± 0.3%, Annexin V+/PI−) and late (28 ± 2.0%, Annexin V+/PI+) apoptosis, as well as in necrosis (16 ± 1.1%, Annexin V−/PI+). The pretreatment with 0.1 mg/mL MJe for 1 h reduced the number of cells undergoing apoptosis (4 ± 0.3% and 13 ± 0.7% of early and late apoptosis, respectively), likewise, in presence of MJe 0.5 mg/mL, it was further reduced to 5 ± 0.2% and 8 ± 0.1%, respectively (Figure 3A,B). The incidence of apoptosis in 6-OHDA-treated cells was confirmed by the results of the caspase 3 activity assay. The exposure to 6-OHDA for 6 h increased caspase 3 activity of SH-SY5Y cells compared to that of unexposed ones by 250 ± 15% (*p* < 0.001; Figure 3C). Pretreatment with MJe (0.1 or 0.5 mg/mL) for 1 h inhibited the activity of caspase 3 induced by 6-OHDA to 140 ± 9 and 123 ± 10%, respectively (*p* < 0.001), while MJe alone, at both tested concentrations, had no effect on this enzymatic activity (Figure 3C).

### 3.4. Protective Effect of MJe on mRNA Levels of Apoptosis-Related Genes Modulated by 6-OHDA

**Figure 4:**
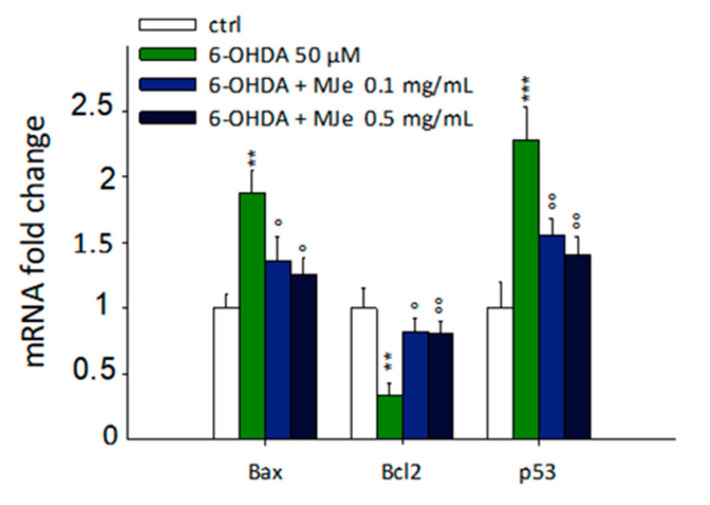
Protective effect of MJe on mRNA levels of apoptosis-related genes modulated by 6-OHDA. SH-SY5Y cells were pretreated with MJe for 1 h and then exposed to 6-OHDA 50 µM for additional 24 h and then assessed with real-time PCR. The 2^–ΔΔCT^ method was employed to calculate the relative quantities of mRNA. Results are expressed as fold change relative to untreated cells. Data, expressed as mean ± SEM, represent the values obtained in three different sets of experiments in triplicate (N = 9). ** *p* < 0.01, *** *p* < 0.001 vs. control; ° *p* < 0.05, °° *p* < 0.01 vs. 6-OHDA 50 µM.

As shown in Figure 4, the incubation of SH-SY5Y cells with 6-OHDA for 24 h significantly enhanced the mRNA levels of the proapoptotic B-cell lymphoma 2 (Bcl-2)-associated X protein (Bax) and p53 genes up to 1.8 ± 0.2- and 2.2 ± 0.3-fold, respectively (*p* < 0.01 and *p* < 0.001), along with decreasing those of the antiapoptotic Bcl-2 up to 3 ± 0.9-fold (*p* < 0.01). These effects were significantly hampered by pre-exposure to MJe at both 0.1 (1.35 ± 0.2-fold and 1.55 ± 0.1-fold down, *p* < 0.05, for Bax and Bcl-2, and 1.2 ± 0.1-fold, *p* < 0.01, for p53) and 0.5 mg/mL concentrations (1.25 ± 0.1-fold, *p* < 0.05, for Bax and 1.25 ± 0.1-fold down and 1.4 ± 0.2-fold, *p* < 0.01 for Bcl-2 and p53) (Figure 4).

### 3.5. Antioxidant Activity of MJe

The antioxidant and radical scavenging properties of MJe were demonstrated using a range of tests as described in the Methods section. As shown in Table 3, the total phenolic content expressed as milligrams of gallic acid equivalents (GAE) per gram of MJe evaluated by Folin–Ciocalteau method was 117.76 ± 4.8. The valuable antiradical activity of MJe shown in the DPPH test and expressed as milligrams of Trolox equivalents (TE) per gram of extract (mgTE/g) was 60.07 ± 4.2, while in the Reducing Power test it was 53.6 ± 2.3 expressed as milligrams of ascorbic acid equivalent (AAE) per gram of MJe (mg AAE/g). Moreover, the obtained values of 3753.7 ± 221.5 µmol TE/g MJe demonstrated high antioxidant capacity of MJe against peroxyl radicals (Table 3).

### 3.6. Effects of MJe on 6-OHDA-Induced SOD and CAT Activities and GSH Content

**Figure 5:**
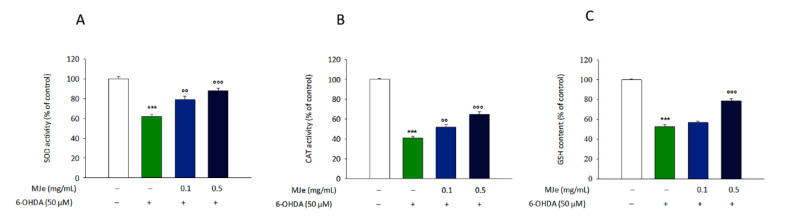
Effect of MJe on biomarkers of 6-OHDA-induced oxidative stress in SH-SY5Y cells. (**A**) Effect of MJe on the activity of SOD in 6-OHDA-treated SH-SY5Y cells; (**B**) Effect of MJe on the activity of CAT in 6-OHDA-treated SH-SY5Y cells; (**C**) Effect of MJe on the GSH levels in 6-OHDA-treated SH-SY5Y cells. Data are showed as the mean ± SEM of three experiments in triplicate (N = 9). *** *p* < 0.001 vs. ctrl; °° *p* < 0.01 and °°° *p* < 0.001 vs. 6-OHDA 50 µM.

We evaluated the levels of catalase and superoxide dismutase activities as well as glutathione content to determine the extent of oxidative stress in the cells. As shown in Figure 5, the treatment of SH-SY5Y cells with 6-OHDA caused a significant decrease in the levels of these biomarkers in comparison to those of control cells (62 ± 2.3, 41 ± 1.7, and 53 ± 2.1% for SOD, CAT, and GSH, respectively, *p* < 0.001). Pretreatment with MJe at both concentrations of 0.1 (62 ± 2.3, 41 ± 1.7, and 53 ± 2.1% for SOD, CAT, and GSH, respectively, *p* < 0.01) and 0.5 mg/mL (88 ± 2.8, 65 ± 2.2, and 79 ± 2.0% for SOD, CAT, and GSH, respectively, *p* < 0.001) for 1 h significantly augmented the activity of both CAT and SOD, along with levels of GSH, indicative of a reduction of oxidative stress (Figure 5).

### 3.7. MJe Reduces Oxidative Stress Induced by 6-OHDA

**Figure 6:**
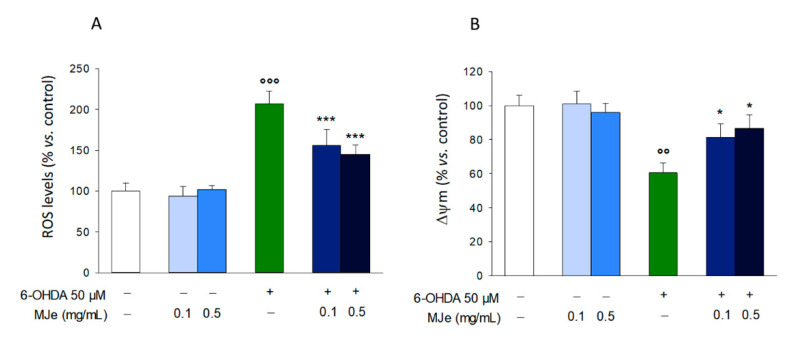
MJe diminished both generation of reactive oxygen species (ROS) and fall of mitochondrial membrane potential (ΔΨm) induced by 6-OHDA. (**A**) ROS accumulation was measured using the fluorescent probe DCFH-DA. (**B)** ΔΨm was assessed using the cationic fluorochrome R123. Results are reported as percentage of the levels detected in untreated cells. Data are displayed as the mean ± SEM of three experiments in triplicate (N = 9). °° *p* < 0.01 and °°° *p* < 0.001 vs. ctrl; * *p* < 0.05 and *** *p* < 0.001 vs. 6-OHDA 50 µM.

The increase of ROS is an acknowledged characteristic of several NDs, including PD. Therefore, we measured the intracellular ROS amount through DCFH-DA. As shown in Figure 6A, the exposure of SH-SY5Y cells to 50 µM 6-OHDA for 6 h caused a significant 2 ± 0.15-fold intracellular ROS accumulation compared to that in the controls (*p* < 0.001; Figure 6A). Pretreatment with MJe at both 0.1 and 0.5 mg/mL concentrations significantly counteracted the increase in ROS caused by 6-OHDA, by 51 ± 0.2 and 63 ± 0.1%, respectively (*p* < 0.001; Figure 6A). Moreover, the exposure of SH-SY5Y cells to 50 µM 6-OHDA for 6 h significantly influenced the ΔΨm that, in comparison to that in control cells, decreased by 39 ± 0.6% (*p* < 0.01). The reduction of ΔΨm evoked by 6-OHDA was prevented by MJe 0.1 and 0.5 mg/mL (81.3 ± 8 and 86.7 ± 7% *p* < 0.05, respectively; Figure 6B).

### 3.8. Protective Effects of MJe on 6-OHDA DNA Damage

**Figure 7:**
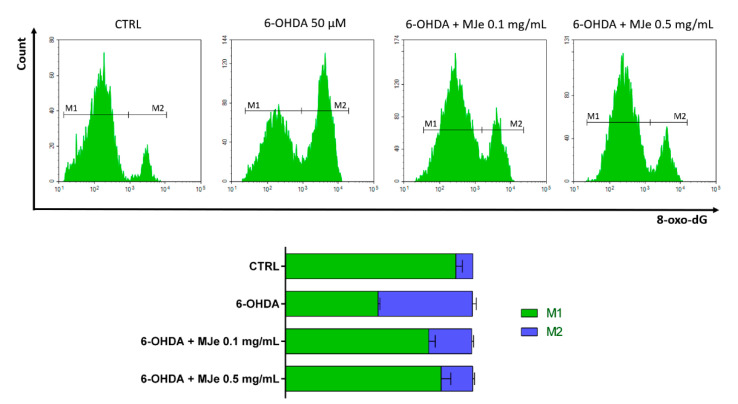
Protective effects of MJe on DNA oxidative damage induced by 6-OHDA. Levels of 8-oxo-dG are measured as emission signals of fluorochrome FITC-labelled avidin. The plots are representative of three independent experiments. The histograms show the percentage ± SEM of healthy cells (non-fluorescent, M1) and the damaged ones (fluorescent, M2) of three separate experiments in triplicate (N = 9).

Levels of 8-oxo-dG were measured to study the efficacy of MJe in containing DNA-oxidative damage using a FITC-conjugated avidin probe (Figure 7). The exposure of SH-SY5Y cells to 6-OHDA 50 µM for 24 h induced DNA oxidation of about 50 ± 2% in comparison to that in control cells. Pretreatment with 0.1 and 0.5 mg/mL of MJe for 1 h before the exposure to 6-OHDA 50 µM (24 h) decreased oxidative DNA damage by 27 ± 1 and 33 ± 1% relative to stressed cells, respectively (Figure 7). The treatment with MJe alone at both concentrations tested did not induce DNA oxidation, since the emission values approximately overlapped those recorded in control cells (data not shown).

### 3.9. MJe Reduces the Production of NO in SH-SY5Y Cells

**Figure 8:**
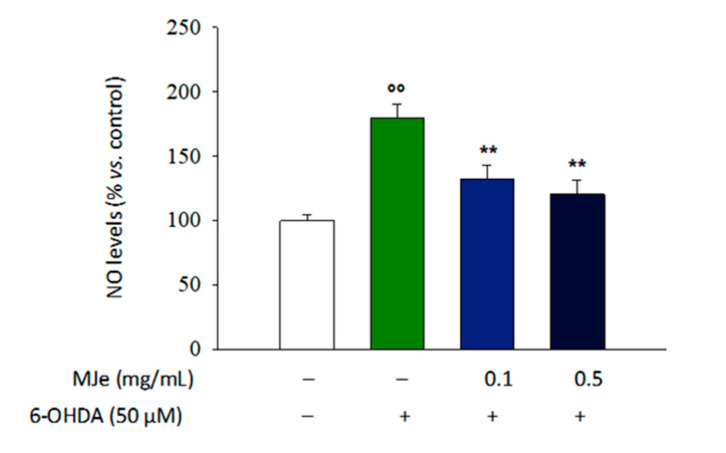
MJe prevented the release of NO induced by 6-OHDA. The levels of NO were measured by a colorimetric assay. Differences in NO production are reported as percentage of NO value detected in treated cells compared to those found in untreated ones. Results are expressed as means ± SEM from three independent experiments in triplicate (N = 9). °° *p* < 0.01 vs. ctrl; ** *p* < 0.01 vs. 6-OHDA 50 µM.

The exposure of differentiated SH-SY5Y cells for 24 h to 6-OHDA brought a 79 ± 5% increase of NO production (*p* < 0.01; Figure 8) that was hindered by pretreatment with MJe for 1 h at both concentrations tested (47 ± 2.5 and 59 ± 2% lower, *p* < 0.01, compared to that in 6-OHDA-treated cells; Figure 8), while MJe alone had no effect on NO production (data not shown).

### 3.10. Protective Effect of MJe on mRNA Levels of Parkinson-Related Genes Modulated by 6-OHDA

**Figure 9:**
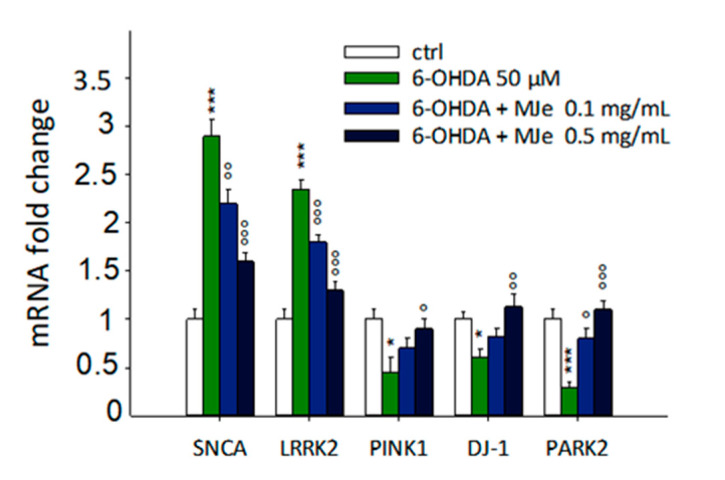
Modulatory effects of MJe on mRNA levels of Parkinson-related genes modulated by 6-OHDA. SH-SY5Y cells were pretreated with MJe for 1 h and then exposed to 6-OHDA 50 µM for additional 24 h. Messenger RNA levels were quantified with real-time PCR, and their relative quantities were calculated through the 2^–ΔΔCT^ method. Results are expressed as fold change relative to untreated cells. Data are expressed as mean ± SEM of three separate experiments in triplicate (N = 9). * *p* < 0.05, *** *p* < 0.001 vs. control; ° *p* < 0.05, °° *p* < 0.01 and °°° *p* < 0.001 vs. 6-OHDA 50 µM.

As shown in Figure 9, the exposure of SH-SY5Y cells to 50 µM 6-OHDA for 24 h significantly enhanced the levels of α-synuclein (SNCA) and leucine-rich repeat kinase 2 (LRRK2) genes up to 2.9 ± 0.2- and 2.3 ± 0.1-fold, respectively (*p* < 0.001), as well as decreased those of phosphatase and tensin homolog (PTEN)-induced putative kinase 1 (PINK1), DJ-1, and parkin (PARK2) up to 2.2 ± 0.15, 1.6 ± 0.1 and 3 ± 0.04-fold down, respectively (*p* < 0.01). SNCA, LRRK2, and PARK2 were significantly modulated by the pre-exposure to MJe at both 0.1 (1.3 ± 0.15-fold down *p* < 0.01 for SNCA, 1.3 ± 0.1-fold down, *p* < 0.001 for LRRK2 and 2.7 ± 0.1-fold *p* < 0.05 for PARK2, relative to 6-OHDA-stressed cells) and 0.5 mg/mL concentrations (1.8 ± 0.1-fold down for both SNCA and LRRK2, and 3.7 ± 0.3-fold for PARK2, *p* < 0.001, respect to 6-OHDA-stressed cells) (Figure 9). Concerning PINK-1 and DJ-1 genes, instead, only the highest concentration of MJe was able to counteract the effect of 6-OHDA (2.0 ± 0.1-fold *p* < 0.05 for PINK1, 1.9 ± 0.2-fold *p* < 0.01 for DJ-1, relative to 6-OHDA-stressed cells).

## 4. Discussion

*Citrus reticulata* (mandarin), originated from Southeast China, is present in Europe under a multitude of varieties and has been studied mainly for its anticancer properties in different in vitro and in vivo models [10]. In these regards, we showed that MJe induces antiproliferative activity in three different anaplastic thyroid carcinoma cell lines, blocking cell cycle in G2/M phase and inducing autophagy, as well as reducing cell migration and affecting metalloproteinase activity [27].

To the best of our knowledge, this study is the first to assess the neuroprotective effect of MJe in 6-OHDA-stressed SH-SY5Y cells, a widely employed cell line to mimic the cellular PD’s environment [28]. Among the most recognized PD models, we chose the 6-OHDA, a neurotoxin inducing depletion of dopaminergic neurons in the substantia nigra pars compacta, given the scientific evidence, both in vitro [29] ad in vivo [30,31], supporting its use.

First, we showed that MJe was able to defend cell viability from damage induced by 6-OHDA, as shown by MTT and trypan blue assays. Moreover, we found that our stressor dramatically increased apoptotic events, as clearly demonstrated by Annexin V/PI staining, an effect that was counteracted by MJe treatment both at 0.1 and 0.5 mg/mL, though to different extent. Apoptosis is regulated by several factors that can push cells towards survival or programmed death. It is also acknowledged that increased ROS levels unleash mitochondrial damage and hence the release of apoptosis inducers [32]. Proapoptotic proteins (i.e., Bax and Bad) and antiapoptotic ones (i.e., Bcl-2 and Bcl-XL) are finely balanced to regulate the fate of each cell in the organism, a stability that comes to an end in cellular degeneration [33]. Moreover, tumor suppressor p53 is sensitive to stress such as DNA damage and hypoxia, being activated by phosphorylation and acetylation, hence inducing cell-cycle arrest and apoptosis [34]. Accordingly, we evaluated the gene expression of the abovementioned factors, finding that Bax and p53 were downregulated by MJe treatment, whereas Bcl-2 was upregulated, in a completely opposite manner of our stressor 6-OHDA. These clearly suggested that their regulation was crucial in the effect of MJe in SH-SY5Y cells. Noteworthy, ROS are known to be able to directly activate the cascade of caspases and hence the whole apoptotic machinery, directly aiming at caspase 3 [35]. Therefore, we also evaluated the involvement of this caspase in the protective effect of MJe, and we witnessed a sharp hindering of its activation compared to the levels observed in control cells, suggesting the role of caspase 3 as a keystone in the overall protective mechanism brought by MJe. As is widely known, 6-OHDA increases ROS levels as a direct result of mitochondrial impairment [36], being one of the possible cellular causes of PD, as well as other neurodegenerative diseases. Consequently, we aimed at evaluating the ROS levels in SH-SY5Y cells after 6-OHDA stress, finding a robust increase of the species, which was blocked by MJe treatment. Moreover, our extract ameliorated ∆ψm, impaired by 6-OHDA injury that, together with the reduction of ROS levels, are clear signals of mitochondrial protection elicited by our extract. Unbalanced ROS production brings its effects at the nucleus level, where oxidative damage on DNA gives birth to oxidized base adducts, among which 8-oxo-dG is a relevant marker of early clinical manifestations of cognitive impairment [37]. Therefore, we evaluated its presence in SH-SY5Y cells after 6-OHDA stress, detecting an increase of the portion of fluorescent cells, and hence carrying this DNA oxidative adduct, whereas MJe was able to lower this effect.

The antioxidant capacity of MJe was also appreciated through the evaluation of typical cellular biomarkers involved in this process, namely SOD, CAT, and GSH. The exposure of SH-SY5Y cells to 6-OHDA decreases cellular antioxidant defense, and flavonoids are acknowledged for hampering it [38]. Here, we found that MJe was able to restore both SOD and CAT activity, two fundamental enzymes that act together to quench oxygen radicals, as well as GSH levels. Interestingly, MJe proved to also be a great antioxidant in abiotic models, where it was able to quench both oxygen (ORAC) and nitrogen (DPPH) radicals along with reducing ferric ions into ferrous ones, effects likely ascribed to the high polyphenol content, as assessed by Folin–Ciocalteu assay and in line with previous reports on *Citrus* extracts [39,40].

Nitric oxide (NO) is another central signaling molecule whose overproduction is acknowledged to be the cause of neuronal impairment, typical of neurodegenerative diseases like PD [41]. Notably, MJe decreased the cellular levels of NO that were drastically increased by 6-OHDA. Furthermore, our extract acted as both antioxidant and reducing agent in different cell-free models, given its high polyphenolic content, reinforcing the results we obtained in vitro. The etiology of PD is not fully understood yet, but scientific evidence shows that mutations in SNCA, PINK1, parkin, DJ-1, and LRRK2 genes are at the basis of familial cases of PD [42]. These encode for proteins that are tightly intertwined in the process of mitophagy and hence in the regulation of neuron viability. After an oxidative stimulus, α-synuclein increases in neurons, forming the so-called Lewy bodies, agglomerates typical of PD. Overexpression of this protein induces an increase of ROS at the cellular level, starting a vicious cycle where α-synuclein induce ROS and vice versa. Gain-of-function mutation of LRRK2 gene brings to an increased susceptibility of neurons to oxidative stress, as widely demonstrated. On the other hand, parkin and its regulator PINK1 are known to be involved in mitochondrial survival and protection against ROS, together with DJ-1 that homodimerizes, becoming another antioxidant neuronal defense [43]. In our study, the oxidative stress induced by 6-OHDA brought an expected sharp increase of both SNCA and LRRK2, an effect that was hampered by MJe treatment. Conversely, the mitochondrial antioxidant machinery, consisting of PINK1/parkin and DJ-1, was negatively affected by the stressor. However, MJe was able to restore the levels of genes encoding the abovementioned proteins and hence improving response against oxidative stress. Our results are in line with previous reports in which natural products were able to hamper neuronal oxidative stress elicited by 6-OHDA, lowering SNCA and LRRK2 expression levels along with increasing those of PINK1/parkin and DJ-1 [44,45].

## 5. Conclusions

Overall, MJe hampered the oxidative stress induced by 6-OHDA treatment in our cellular model, contemporarily targeting the mitochondria, nucleus, and cytoplasm, protecting these compartments by ROS overproduction and increasing the survival rate, along with blocking apoptotic machinery. From a molecular point of view, MJe restored the gene expression of factors linked to mitochondrial functionality, acknowledged to be crucial in PD clinical outcomes, whose balance was impaired by 6-OHDA. Therefore, we suggest the great validity of MJe in facing oxidative-based diseases, such as PD, that needs to be also proven in more complex models to corroborate our statements.

## Figures and Tables

**Table 1:** Oligonucleotide primers used for real-time PCR.

Gene Product	NCBI Reference Sequence	Primer Sequence
p53	NM_000546.6	Forward: 5′-GTGTGGAGTATTTGGATGAC-3′ Reverse: 5′-ATGTAGTTGTAGTGGATGGT-3′
Bax	NM_138764.5	Forward: 5′-GGACGAACTGGACAGTAACATGG-3′ Reverse: 5′-GCAAAGTAGAAAAGGGCGACAAC-3′
Bcl-2	NM_000657.3	Forward: 5′-ATCGCCCTGTGGATGACTGAG-3′ Reverse: 5′-CAGCCAGGAGAAATCAAACAGAGG-3′
SNCA	NM_000345.4	Forward: 5’-TGACAAATGTTGGAGGAGCA-3’ Reverse: 5’-TGTCAGGATCCACAGGCATA-3’
LRRK2	NM_198578.4	Forward: 5’-TCAGCTTGTTGTTGGACAGC-3’ Reverse: 5’-ACTGCGTGAGGAAGCTCATT-3’
PINK1	NM_032409.3	Forward: 5’-ACGTTCAGTTACGGGAGTGG-3’ Reverse: 5’-GGCTAGTCAGGAGGGAAACC-3’
DJ-1	NM_007262.5	Forward: 5’-GGGTGCAGGCTTGTAAACAT-3’ Reverse: 5’-GGACAAATGACCACATCACG-3’
PARK2	NM_004562.3	Forward: 5’-CTGACACCAGCATCTTCCAG-3’ Reverse: 5’-CCAGTCATTCCTCAGCTCCT-3’
β-Actin	NM_001101.5	Forward: 5′-TTGTTACAGGAAGTCCCTTGCC-3′ Reverse: 5′-ATGCTATCACCTCCCCTGTGTG-3′

**Table 2:** Quantitative determination of the identified compounds.

		mg/g (Dried Extract)	
Peak	Compounds	Mean	SD
1	Vicenin-2	55.2	2.5
2	Lucenin-2 4′-methyl ether	21.5	1.7
3	Orientin4′methylether	1.4	0.21
4	Eriocitrin	8.2	1.6
5	Narirutin	47.8	1.9
6	Hesperidin	353.6	20.5
7	Sinensetin	2.0	0.3
8	Tangeretin	6.4	0.5
9	Nobiletin	17.3	1.4

**Table 3:** Antioxidant activity of MJe evaluated by abiotic assays. Results are reported as mean ± SEM of three experiments performed in triplicate and expressed in standard equivalent/g of dried extract (N = 9).

Folin-Ciocalteau (mg GAE/g)	117.76 ± 4.8
DPPH (mg TE/g)	60.07 ± 4.2
Reducing Power (mg AAE/g)	53.6 ± 2.3
ORAC (µmol TE/g)	3753.72 ± 221.5

## Data Availability

Data is contained within the article.
